# Mutations Status of Chemokine Signaling Pathway Predict Prognosis of Immune Checkpoint Inhibitors in Colon Adenocarcinoma

**DOI:** 10.3389/fphar.2021.721181

**Published:** 2021-10-14

**Authors:** Anqi Lin, Wentao Xu, Peng Luo, Jian Zhang

**Affiliations:** Department of Oncology, Zhujiang Hospital, Southern Medical University, Guangzhou, China

**Keywords:** chemokine, immune checkpoint inhibitors, mutations, colon adenocarcinoma, tumor microenvironment

## Abstract

In recent years, tumor immunotherapy has become an important treatment program and popular research focus. However, the use of immune checkpoint inhibitors (ICI) in the treatment of colorectal cancer still has limitations due to the current markers only being able to predict the prognosis of a small number of patients. As the chemokine signaling pathway can promote the anti-tumor response of the immune system by recruiting immune cells, we explored the relationship between mutations in the chemokine signaling pathway and the prognosis of colon adenocarcinoma (COAD) patients receiving ICI treatment. To analyze the relationship between chemokine mutation status and the prognosis of patients receiving ICI treatment, clinical and mutation data, with immunotherapy, for a COAD cohort was obtained from “cbioportal.” Then, combining this with COAD cohort data from The Cancer Genome Atlas (TCGA) database, the panorama of gene mutation, immunogenicity, and difference in tumor microenvironment (TME) of chemokine pathways with different mutation statuses were analyzed. High-mut status has been proved to be a prognostic indicator of COAD patients receiving ICI treatment by Univariate and Multivariate Cox regression analysis. CIBERSORT analysis showed that the infiltration degree of M1 macrophages, neutrophils, and activated natural killer (NK) cells was higher in those with high-mut status. Immunogenicity of the high-mut group was also significantly increased, with the mutation number of tumor mutation burden (TMB), neoantigen load (NAL), DNA damage repair (DDR) pathway and microsatellite instability biomarker (MSI-H) being significantly higher. In this study, we found that the mutation state of chemokine pathways is closely associated with the prognosis of COAD patients undergoing ICI treatment. The higher number of TMB, NAL, and DDR mutations and inflammatory TME, may be the mechanism of behind a better prognosis. This discovery provides a possible idea for ICI therapy of COAD.

## Introduction

Colorectal cancer is a malignant tumor of the gastrointestinal tract, which originates in the colon or rectum. By 2020, the incidence of colorectal cancer ranked third in the world (accounting for 10% of all malignant tumors), while the mortality rate ranked second (9.4%) (Sung et al., 2021). Colon adenocarcinoma (COAD) is the most common malignant tumor of the large intestine (up to 95%) (Fleming et al., 2012). In the last few years, immunotherapy, especially immune checkpoint inhibitors (ICI) treatment, has become an important cancer treatment modality and popular research focus. However, the research has not yet provided a breakthrough in the immunotherapy of colorectal cancer. At present, mismatch repair deficient (dMMR)/microsatellite instability high (MSI-H) is the preferred biomarker for ICI treatment of COAD, and the effect of treatment with ICIs on these patients is remarkable. However, for patients with mismatch repair proficient (pMMR), microsatellite stable (MSS) or microsatellite instability low (MSI-L) status, it is unable to effectively predict the efficacy of ICI treatment (Ganesh et al., 2019). Only 15% of patients with colorectal cancer are dMMR-MSI-H (Ganesh et al., 2019). In addition, although some studies have found that TMB and POLE P286R mutations can be used as new biomarkers (Ahn et al., 2016; Schrock et al., 2019; Loupakis et al., 2020), they also have some limitations. For example, the research on TMB is only based on dMMR and MSI-H patients (Schrock et al., 2019; Loupakis et al., 2020), and there is no difference in immune spectrum between tumor cells and tumor-infiltrating lymphocytes (TILs) between POLE-MT and POLE-WT (Ahn et al., 2016). As a result, it is imperative to find new and reliable biomarkers to predict the efficacy of treatment with ICI.

Chemokines are small cytokines or signal proteins that induce the chemotaxis and recruitment of nearby immune cells, which enhances the anti-tumor ability of the immune response. For example, CXCR5 can activate B cells (Letourneur et al., 2020), and CXCL9/10/11 can induce NK and T cells to become enriched in the region of the tumor cells (Letourneur et al., 2020). In addition, chemokines can also be used as biomarkers to predict the prognosis of patients with various cancers, being treated with ICIs. For example, CXCR3 can be used as an important biomarker for predicting the effect of treatment with anti-PD-1 in melanoma patients (Chow et al., 2019). What is more, some studies have found that the high expression of CXCL9 and CXCL10 can effectively improve the prognosis of anti-PD-1/anti-CTLA-4 treatment (House et al., 2020). However, there is a lack of research on the relationship between chemokine pathway mutations and prognosis for patients undergoing ICI treatment.

To explore the relationship between chemokine pathway mutation status and ICI treatment prognosis, we analyzed the COAD data sets in TCGA and MSKCC, and comprehensively analyzed whether chemokine pathway high-mut status was related to a curative effect of treatment with ICI. We also used bioinformatics to determine a potential mechanism, from a genetic to cellular level.

## Materials and Methods

### ICI-Treated Cohort, and TCGA-COAD Cohort

We downloaded clinical data and mutation data, with immunotherapy prognosis, for a COAD cohort from ‘cbioportal’ (Samstein et al., 2019), and recorded it as “ICI-treated cohort.” We also downloaded clinical data and somatic mutation data of a COAD cohort from The Cancer Genome Atlas (TCGA) database using the “TCGAbiolinks” R package (Colaprico et al., 2016), and recorded it as “TCGA-COAD cohort.” These two cohorts were used for the subsequent analyses. To validate our results, we also downloaded the non-immunotherapy COAD, immune therapy ESCA (esophageal carcinoma), NSCLC (non-small cell lung cancer), and SKCM (skin cutaneous melanoma) cohorts from “cbioportal” as control sets (Van Allen et al., 2015; Zehir et al., 2017; Samstein et al., 2019).

### Non-Synonymous Mutations of Chemokine and DDR Pathways

We downloaded from the molecular signatures database (MsigDB) gene sets of the chemokine pathway and DDR pathway (Liberzon et al., 2011), and downloaded the details of chemokine pathway gene set from “https://www.genome.jp/pathway/hsa04062” (Supplementary Table S2). Whether the corresponding gene has non-synonymous mutations was determined from the non-synonymous mutation data of each patient. Then, on the basis of the median counts of non-synonymous mutations in the chemokine pathway, patients were assigned to the high-mut or low-mut group, and the difference of non-synonymous mutations in the DDR pathway between the high-mut and low-mut groups was studied.

### Gene Set Enrichment Analysis (GSEA) Analysis

In the R programming language, we used the “TCGAbiolinks” package to download the raw count gene expression data of COAD, and then analyze the difference between high-mut and low-mut by “edgeR” package (Robinson et al., 2010). The “clusterProfiler” package was used for the gene annotation enrichment analysis (Yu et al., 2012), and *p* < 0.05 was regarded as a significant difference in Reactome, Kyoto encyclopedia of genes and genomes (KEGG), and gene ontology (GO) terms. The gene set of GSEA enrichment analysis came from the MSigDB database (Subramanian et al., 2005).

### TME Analysis

We imported the expression data of TCGA-COAD into CIBERSORT Web Portal for evaluation (Newman et al., 2015), analyzing the proportion of 22 types of immune cells in the tumor microenvironment (TME). The immune checkpoint molecules and immune-related scores are from Thorsson et al. (2018). The MANTIS score, a prediction of microsatellite instability (MSI) (Bonneville et al., 2017), was used to assign patients as either MSI-L or MSI-H (MSI low or high, respectively). The ESTIMATE algorithm was used to calculate the immune score (Yoshihara et al., 2013).

### Connectivity Map (CMap) Analysis

We analyzed the difference in gene expression between the high and low-mut groups of TCGA, and converted the gene ID into GPL96 format. Then, we exported the first 500 different genes which were up- or down-regulated to a grp file, and carried out a cMap analysis in Build 02 (https://portals.broadinstitute.org/cmap/) (Lamb et al., 2006).

### Statistical Analysis

To identify the influence of chemokine pathway mutations on the clinical prognosis of COAD after ICI treatment, we used the Univariate and Multivariate Cox regression analysis, and to calculate the 95% confidence intervals (CI) and hazard ratio (HR). A Mann-Whitney U test was used to compare TMB and NAL in high-mut and low-mut patients, and the top 20 genes with the mutation frequency were compared by Fisher’s exact test. Kaplan-Meier (KM) survival curves were used to evaluate the relationship between chemokine pathway mutation and overall survival (OS) of COAD patients treated with ICI, and statistical differences were evaluated by log-rank *p* values. *p* < 0.05 was supported to be a statistically significant difference. All the tests and visualization processes were completed in R software.

## Result

### Chemokine Pathway Mutation Status can Be Used as a Predictor of Immunotherapy for COAD

**FIGURE 1:**
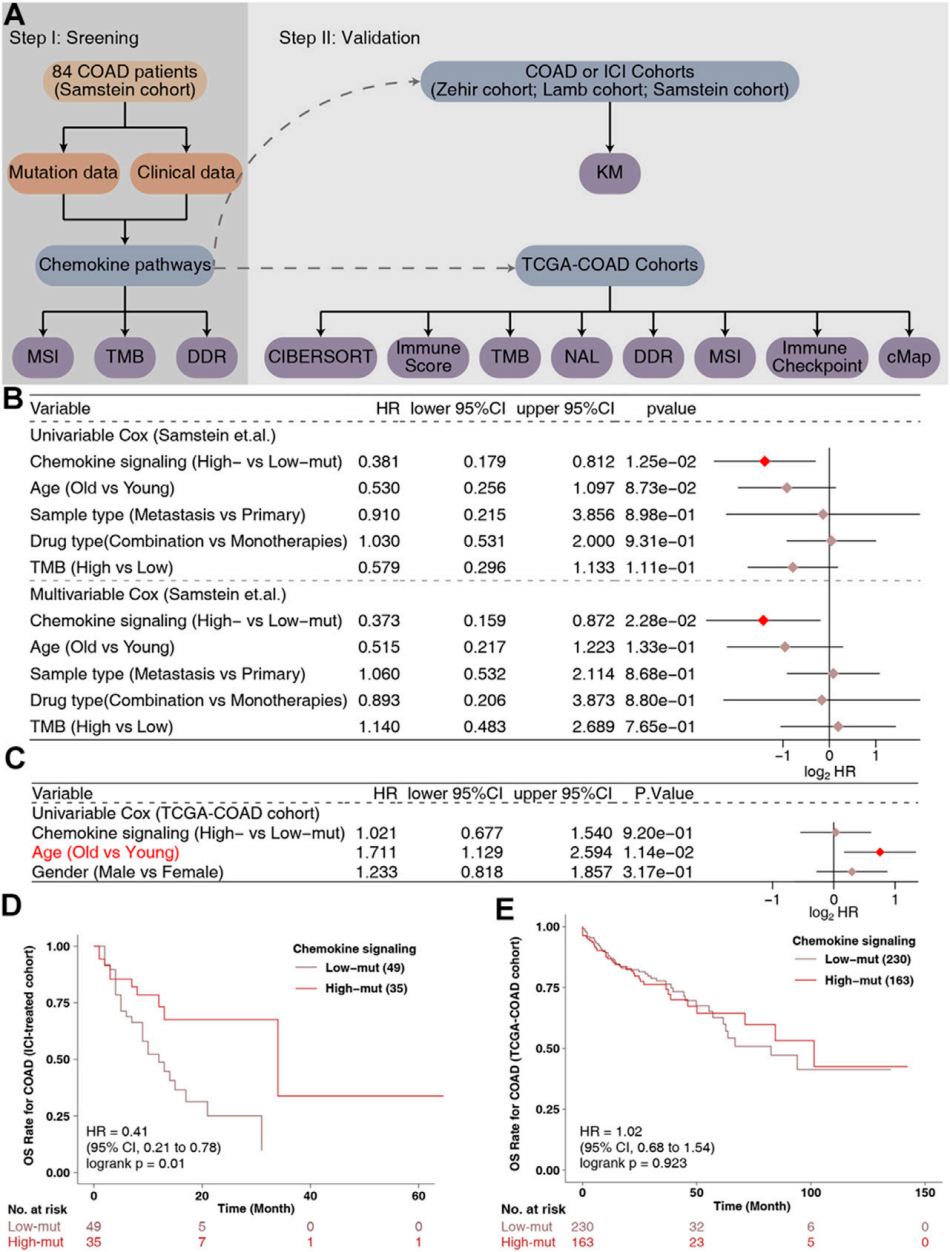
The predictive value of clinical characteristics and the mutation status of the chemokine signaling pathway in terms of ICI efficacy. **(A)** Flowchart of the data processing in this study. Forest plot of the results of the Univariate and Multivariate Cox regression analyses in the **(B)** ICI-treated cohort (Samstein et al., 2019) and **(C)** TCGA-COAD cohort. The main portion of the forest plot presents the hazard ratios (HRs) and 95% confidence intervals (95%CIs), and red dots indicate *p* values <0.05. The HR indicates whether the factors are predictors of favorable (HR < 1) or poor (HR > 1) outcomes. **(D)** KM survival curves for OS in 84 COAD patients from the ICI-treated cohort (Samstein et al., 2019). **(E)** KM survival curves for OS in 393 COAD patients from the TCGA-COAD cohort.

According to the non-synonymous mutations in the chemokine pathway of each patient in the ICI-treated cohort, we divided the patients into high-mut and low-mut groups. A univariate Cox regression analysis showed that clinical features such as age (old vs. young) and sample type (metastatic vs. primary) were not related to the survival of patients treated with ICI patients, but that the chemokine pathway in the high-mut group could be used as a protective factor for COAD patients receiving immunotherapy (Figure 1B). Multivariate Cox regression analysis further showed that high-mut could be used as an independent protective factor for immunotherapy in patients with COAD (Figure 1B). In the ICI-treated cohort, 84 COAD patients were divided into high-mut and low-mut groups for survival analysis to verify whether chemokine pathway mutations can effectively predict the prognosis of these patients (Figure 1D). It was found that high-mut was associated with longer OS (Logrank test, HR = 0.41, 95% CI [0.21–0.78], *p* = 0.01). Interestingly, in the TCGA-COAD cohort, chemokine pathway mutations cannot be used as a prognostic indicator for COAD patients (Figures 1C,E), which indicates that chemokine pathway mutations cause other mechanisms which affect the sensitivity of COAD patients to immunotherapy.

### Differences in Gene Mutations and Clinical Characteristics Between High and Low Mutation Groups

**FIGURE 2:**
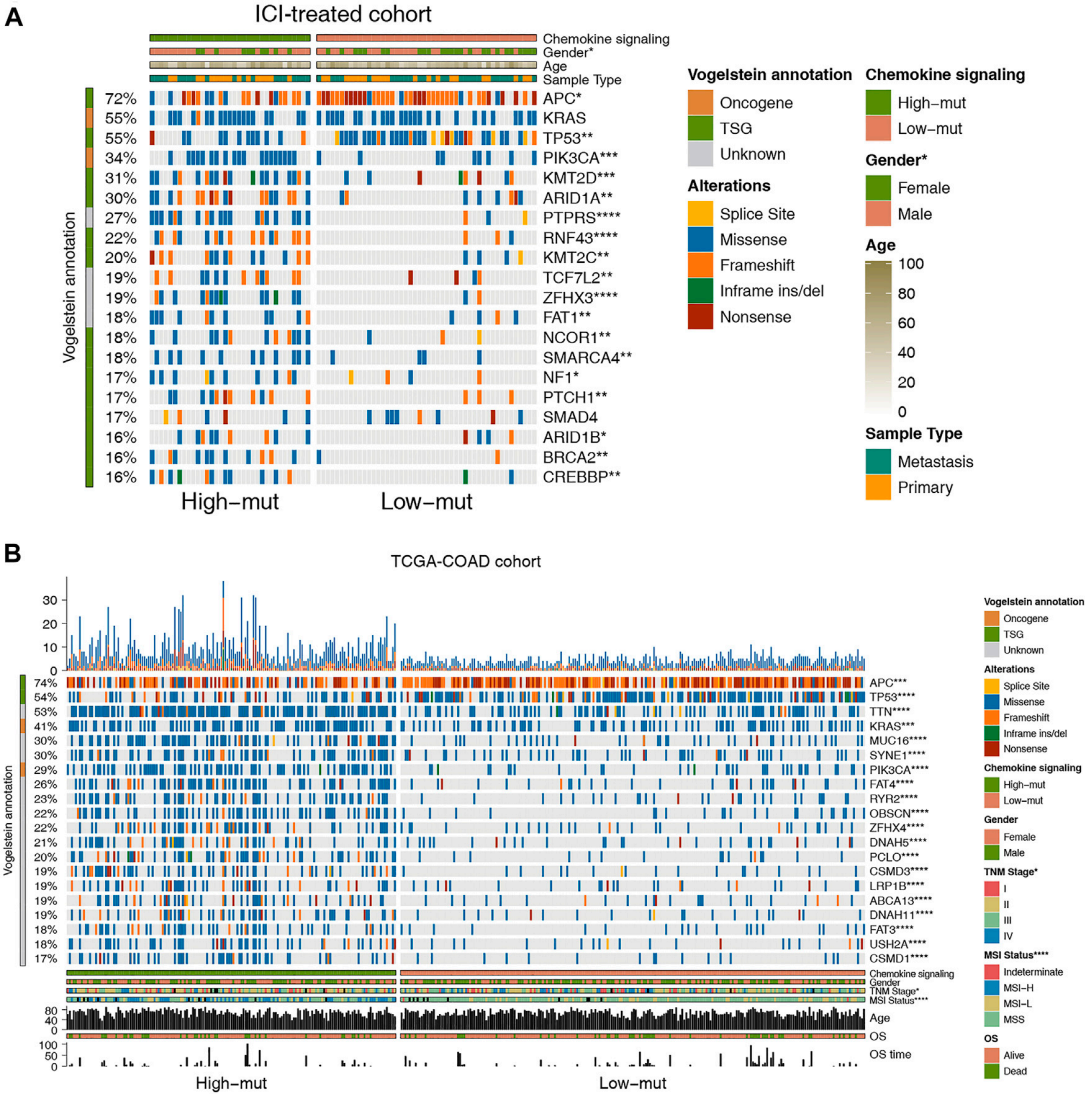
Genomic profiles of COAD patients in the ICI-treated cohort (Samstein et al., 2019) **(A)** and the TCGA-COAD cohort **(B)**. The 20 genes with the highest mutation frequencies and corresponding clinical information are shown in the figure.

**FIGURE 3:**
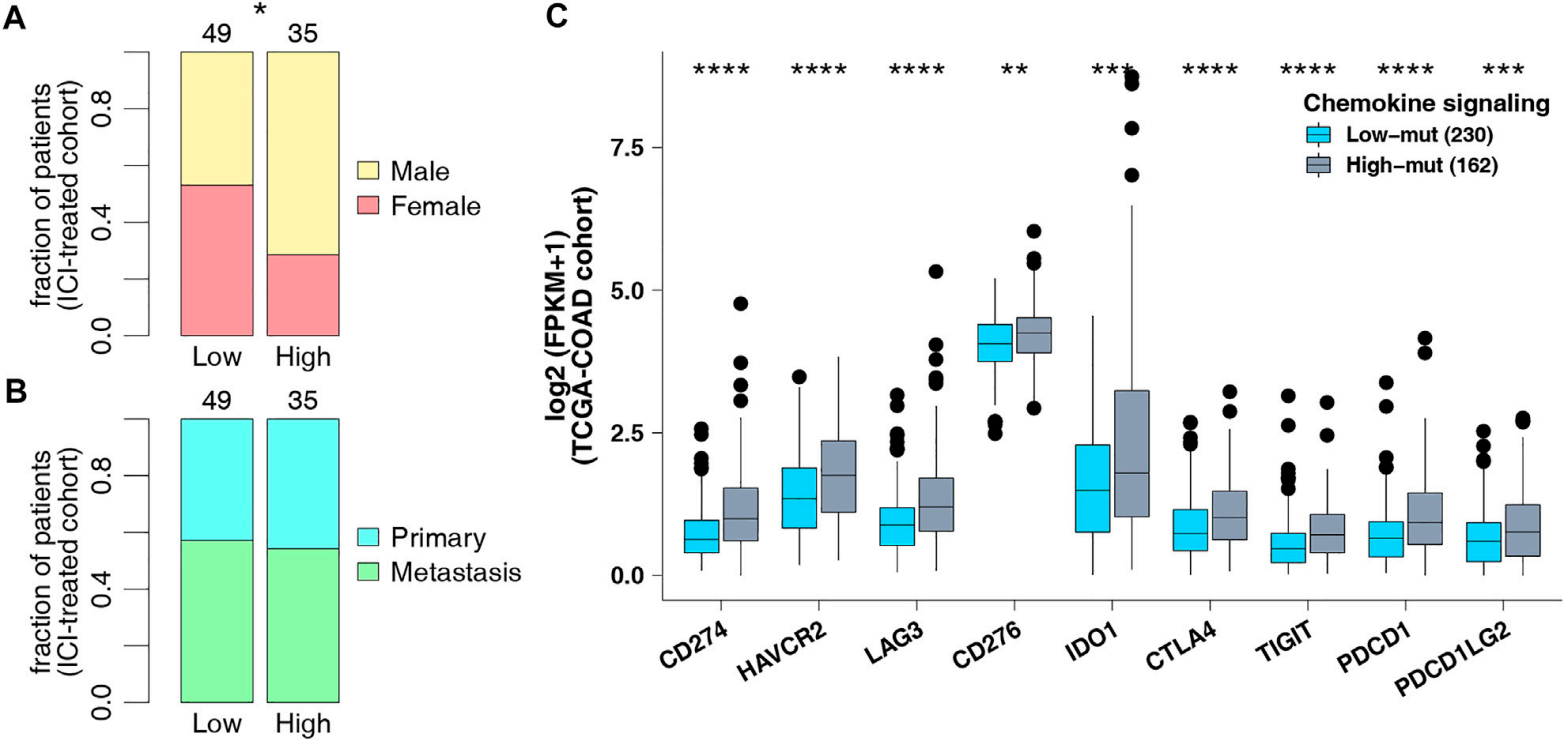
**(A)** Comparison of sex in the ICI-treated cohort. **(B)** Comparison of sample types in the ICI-treated cohort. **(C)** Comparison of the expression of immune checkpoint genes in the TCGA-COAD cohort.

To explore the difference in somatic mutation frequency between high-mut and low-mut groups, we analyzed the nonsensical somatic mutations in the ICI-treated and TCGA-COAD cohorts, and drew the panorama of gene mutation for the top 20 somatic mutations (Figure 2). In the ICI cohort, with the exception of APC, TP53, and KRAS, the mutation frequency of mutant genes in the high-mut group increased significantly (Figure 2A). These include PIK3CA (0.54:0.18, *p* < 0.05), KMT2D (0.51:0.16, *p* < 0.05), ARID1A (0.49:0.16, *p* < 0.05), PTPRS (0.54:0.06, *p* < 0.05), and RNF43 (0.43:0.06, *p* < 0.05). In the TCGA-COAD cohort, with the exception of APC and TP53, the mutation frequency of mutant genes in the high-mut group up-regulated significantly (*p* < 0.05; Figure 2B). These include TTN (0.72:0.39, *p* < 0.05), KRAS (0.51:0.34, *p* < 0.05), MUC16 (0.48:0.18, *p* < 0.05), SYNE1 (0.41:0.22, *p* < 0.05), and PIK3CA (0.47:0.16, *p* < 0.05). Only two of the top 20 mutant genes in the two cohorts were proto-oncogenes (KRAS and PIK3CA). We then analyzed the co-occurrence and mutual exclusion of the top 20 mutant genes in each cohort (Supplementary Figure S1). Also of note, in the ICI cohort the proportion of male patients in the high-mut group was significantly higher than that in the low-mut group (*p* < 0.05; Figure 3A), however, The sample type did not show significant statistical difference (*p* > 0.05; Figure 3B). In the TCGA-COAD cohort, although previous analyses showed that gender can be used as an index of prognosis and survival (Figure 1C), there was no statistically significant difference between the high-mut and low-mut groups (*p* > 0.05). However, there were significant differences in the clinical stage and MSI status of tumors (*p* < 0.05; Figure 2B), which suggests the mutation of chemokine pathways is not related to gender.

As checkpoint molecules are important targets for ICI treatment, we analyzed the difference in expression of checkpoint molecules between the high-mut and low-mut groups in the TCGA cohort. We found that the expression of checkpoint molecules in the high-mut group was significantly higher than the low-mut group (*p* < 0.05; Figure 3C).

### Tumor Immunogenicity May Be the Reason why the Chemokine Pathway can Be Used as a Predictor

**FIGURE 4:**
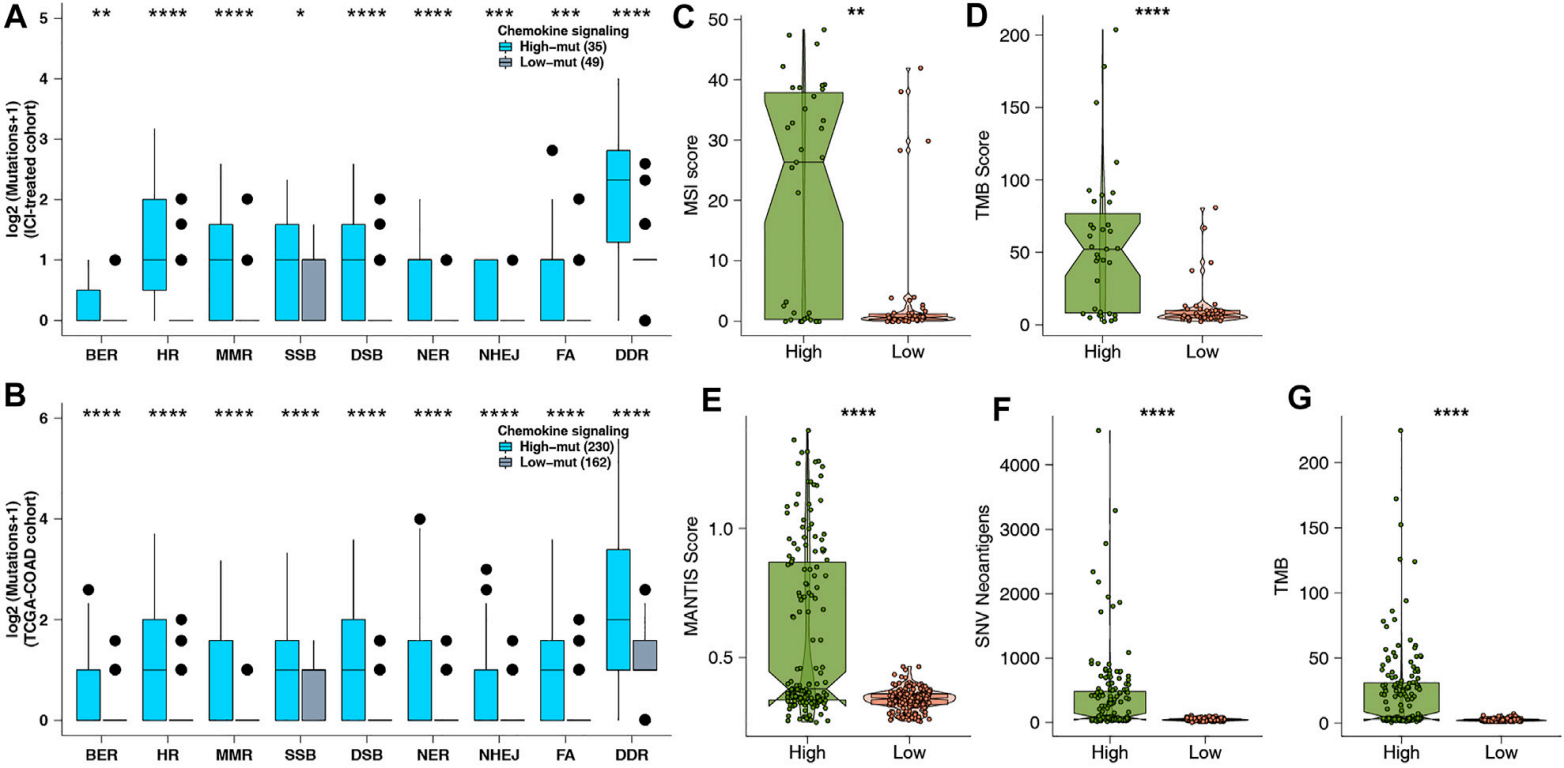
**(A)** Comparison of DDR signaling alterations between the high-mut and low-mut groups in the ICI-treated cohort (Samstein et al., 2019). **(B)** Comparison of DDR signaling alterations between the high-mut and low-mut groups in the TCGA-COAD cohort. **(C)** Comparison of MSI between the high-mut and low-mut groups in the ICI-treated cohort (Samstein et al., 2019). **(D)** Comparison of TMB between the high-mut and low-mut groups in the ICI-treated cohort (Samstein et al., 2019). **(E)** Comparison of MSI between the high-mut and low-mut groups in the TCGA-COAD cohort. **(F)** Comparison of NAL between the high-mut and low-mut groups in the TCGA-COAD cohort. **(G)** Comparison of TMB between the high-mut and low-mut groups in the TCGA-COAD cohort.

Genome instability is one of the important theoretical foundations of immunotherapy, and DNA damage repair genes hold an important role in maintaining the stability of the genome. To further explore the potential mechanism of predicting ICI efficacy in the high-mut group, we downloaded eight gene sets related to DDR, from MSigDB to study whether the number of DDR pathway mutations in the high-mut group was different from that in the low-mut group. We found that the number of non-synonymous mutations in the DDR pathway in patients with high-mut was significantly higher than that in patients with low-mut, in both the ICI-treated, and TCGA-COAD cohorts (*p* < 0.05; Figures 4A,B). In addition, as MSI, TMB, and NAL are predictors of suitability for treatment with ICI, we studied the relationship between each of them and their high-mut/low-mut status. We found that in the ICI cohort, MSI scores of high-mut patients were significantly higher than that of low-mut patients (*p* < 0.05; Figure 4C). Similarly, in the TCGA-COAD cohort, MANTIS scores of the high-mut group were significantly higher than that of the low-mut group (*p* < 0.05; Figure 4E). In both ICI-treated and TCGA-COAD cohorts, the TMB of high-mut patients was significantly higher than that of the low-mut patients (*p* < 0.05; Figures 4D,G). In addition, in the TCGA cohort, NAL of the high-mut group was significantly higher than that of the low-mut group (*p* < 0.05; Figure 4F).

### TME Could Explain the Sensitivity of the High-Mut Group to Immunotherapy

**FIGURE 5:**
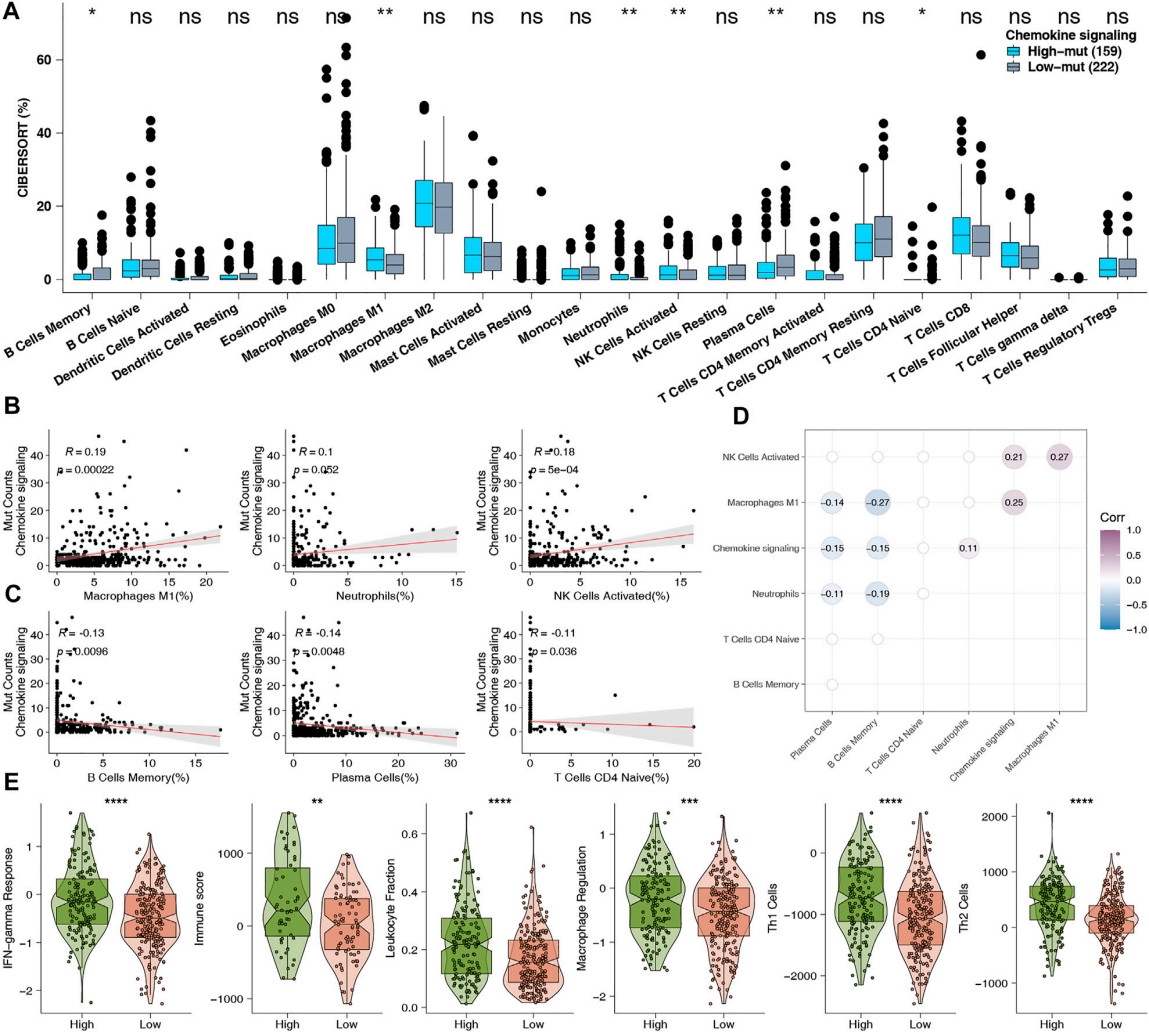
**(A)** Comparison of the proportions of 22 types of immune cells estimated by the CIBERSORT method between high-mut and low-mut tumors in the TCGA-COAD cohort. **(B)** The correlation between the proportion of each higher degree of infiltration immune cells and the counts of DDR pathway mutations in the high-mut group of TCGA-COAD cohort. **(C)** The correlation between the proportion of each lower degree of infiltration immune cells and the counts of DDR pathway mutations in the high-mut group of TCGA-COAD cohort. **(D)** The difference in proportion of 22 types of immune cells. **(E)** Comparison of immune related scores between high-mut and low-mut tumors in the TCGA-COAD cohort. The immune related scores were IFN-gamma Response, Immune, Leukocyte Fraction, Macrophage Regulation, Th1 Cell, and Th2 Cell scores.

The tumor immune microenvironment is tightly correlated with tumor growth, occurrence, metastasis, and anti-tumor immune response, as well as being important as a predictor of the curative effect of treatment with ICI. Therefore, we investigated the underlying mechanisms behind how high-mut status can predict the curative effect of ICI, with regard to TME. First, we analyzed the expression data of the TCGA-COAD cohort by CIBERSORT, and compared the infiltration status of 22 types of immune cells between the high-mut and low-mut groups (Figure 5A). It was found that the infiltration degree of M1 macrophages, neutrophils, and activated NK cells in the high-mut group was higher than that of the low-mut group (*p* < 0.05). In contrast to this, the infiltration degree of memory B cells, plasma cells, and immature T cells was higher in the low-mut group (*p* < 0.05). We analyzed the correlation between the counts of non-synonymous mutations in the chemokine pathway and these six significantly different immune cells. The results demonstrated that M1 macrophages (R = 0.19, *p* < 0.05) and activated NK cells (R = 0.18, *p* < 0.05) were positively correlated with the number of mutations (Figure 5B), while memory B cells (*p* < 0.05, R = -0.13), plasma cells (*p* < 0.05, R = -0.14). and naive CD4 T cells (*p* < 0.05, R = -0.11) were negatively correlated with the number of mutations (Figure 5C). In addition, there was no statistically significant correlation but a positive trend was observed between neutrophils and mutation number (*p* = 0.052, R = 0.1). We also analyzed the correlation among these six immune cells (Figure 5D) and performed cMap analyses to explore which drugs can promote or inhibit chemokine mutation of COAD (Supplementary Table S1). In addition, we calculated the related immune scores (IFN-γ response, immune, leukocyte fraction, macrophage regulation, TH1 cell, and TH2 cell scores), and found that the immune related scores of the high-mut group were significantly higher than those of the low-mut group (*p* < 0.05; Figure 5E).

### GSEA Analysis Revealed the Mechanism Underlying the Improved Responses and Prognosis of High-Mut Patients Treated With ICI

**FIGURE 6:**
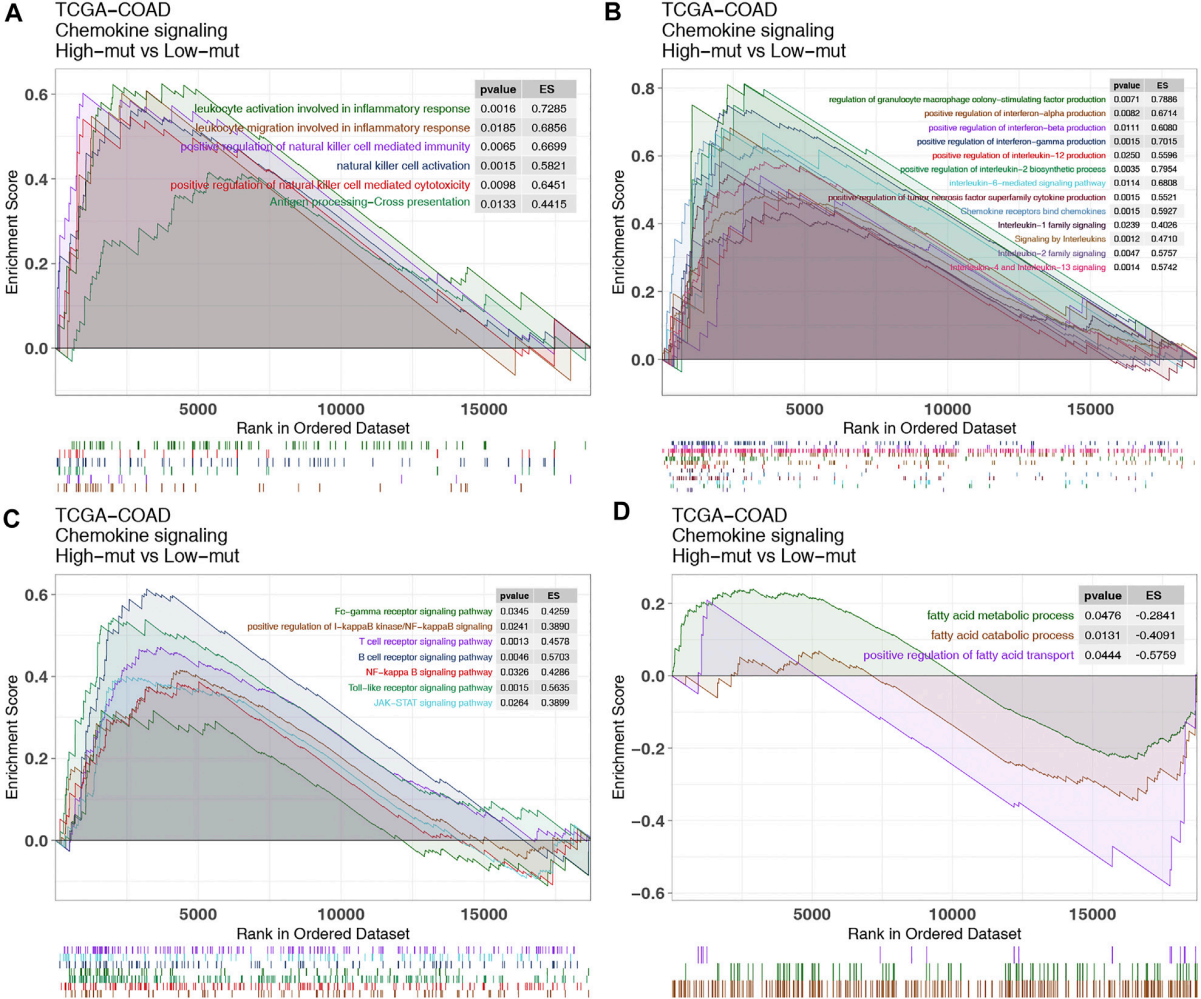
Differentially enriched biological functions between high-mut and low-mut tumors in the TCGA-COAD cohort (identified by GSEA). Differences in immune cells **(A)**, cytokines **(B)**, classical immune related pathways **(C)** and exhaustion-related factors **(D)** between high-mut and low-mut tumors in the TCGA-COAD cohort (identified by GSEA).

Subsequently, we used GSEA to study the functional gene sets enriched in the high-mut and low-mut groups. It was found that some pathways related to the killing function of immune cells were upregulated in the high-mut group (Figure 6A). This included leukocyte migration involved in inflammatory response, natural killer cell activation, antigen processing-Cross presentation, and so on. In addition, some cytokine related pathways (such as tumor necrosis factor, interferon, interleukin, colony stimulating factor, and chemokine related pathways) were also significantly enriched in the high-mut group (Figure 6B). This is evident as positive regulation of interferon-alpha production, positive regulation of interleukin-2 biosynthetic process, interleukin-2 family signaling, interleukin-6-mediated signaling pathway, signaling by interleukins, chemokine receptors bind chemokines, and so on. The activity of signaling pathways closely related to cellular immune response was also up-regulated in the high-mut group (Figure 6C), such as JAK-STAT, Toll-like receptor, B cell receptor, T cell receptor, and the Fc-gamma receptor signaling pathway. In contrast, the pathways related to immune depletion were enriched in the low-mut group (Figure 6D), such as fatty acid metallic process, the positive regulation of fatty acid transport, and so on.

### Chemokine Mutation Status Cannot Only Be Used as a Prognostic Indicator in COAD Patients

**FIGURE 7:**
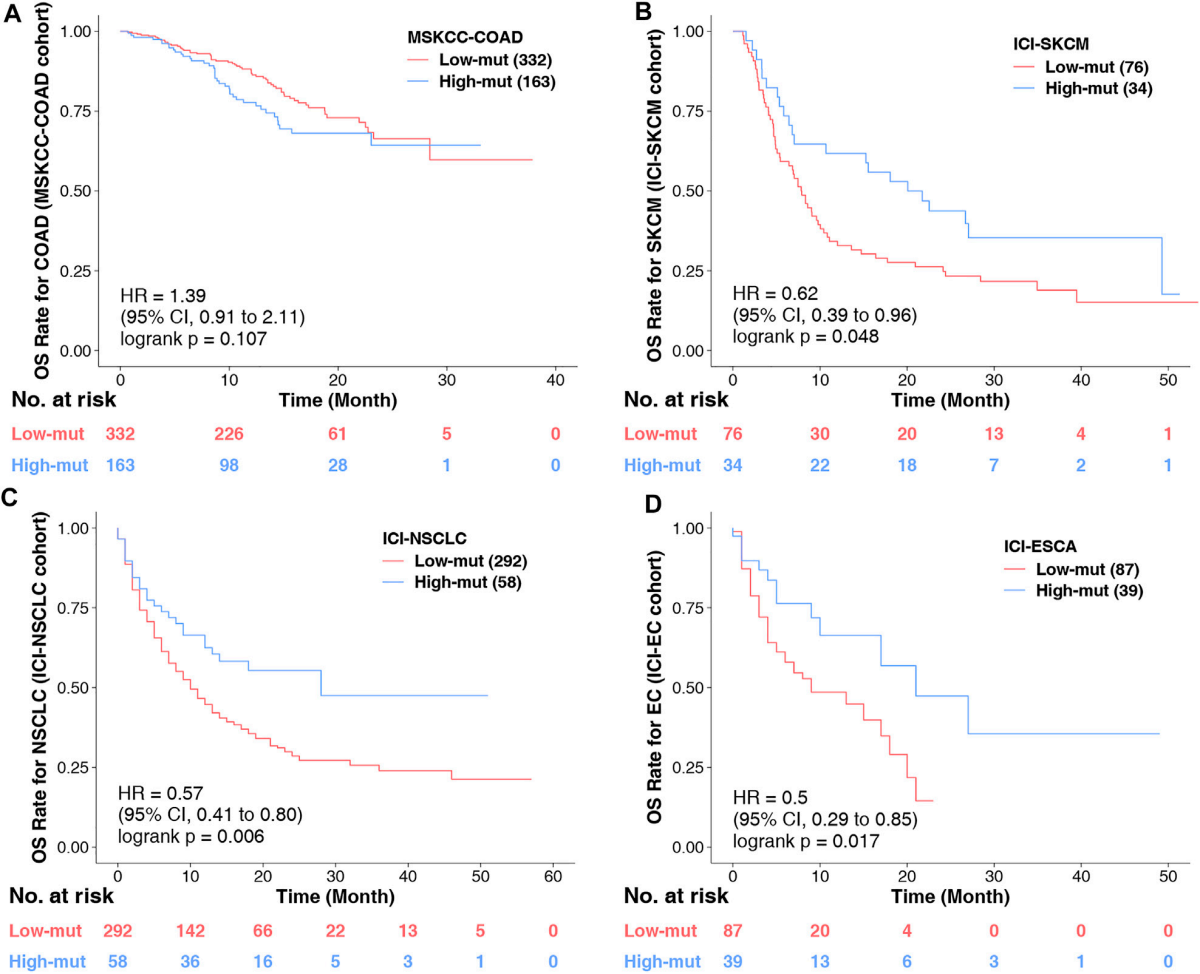
**(A)** KM survival curves for OS in 495 COAD patients from the MSKCC-COAD cohort (Zehir et al.). **(B)** KM survival curves for OS in 110 COAD patients from the ICI-SKCM cohort (Lamb et al.). **(C)** KM survival curves for OS in 350 COAD patients from the ICI-NSCLC cohort (Samstein et al., 2019). **(D)** KM survival curves for OS in 126 COAD patients from the ICI-ESCA cohort (Samstein et al., 2019).

Finally, we conducted KM analyses on other COAD non-immunotherapy cohorts, and other tumor immunotherapy cohorts. It was found that chemokine mutations cannot predict the prognosis of COAD without immunotherapy (Figure 7A), but can predict the prognosis of other tumors with immunotherapy (Figures 7B–D). These conclusions further confirm our findings and suggest that chemokine mutations play a role not only in COAD, and is deserving of further study.

## Discussion

**FIGURE 8:**
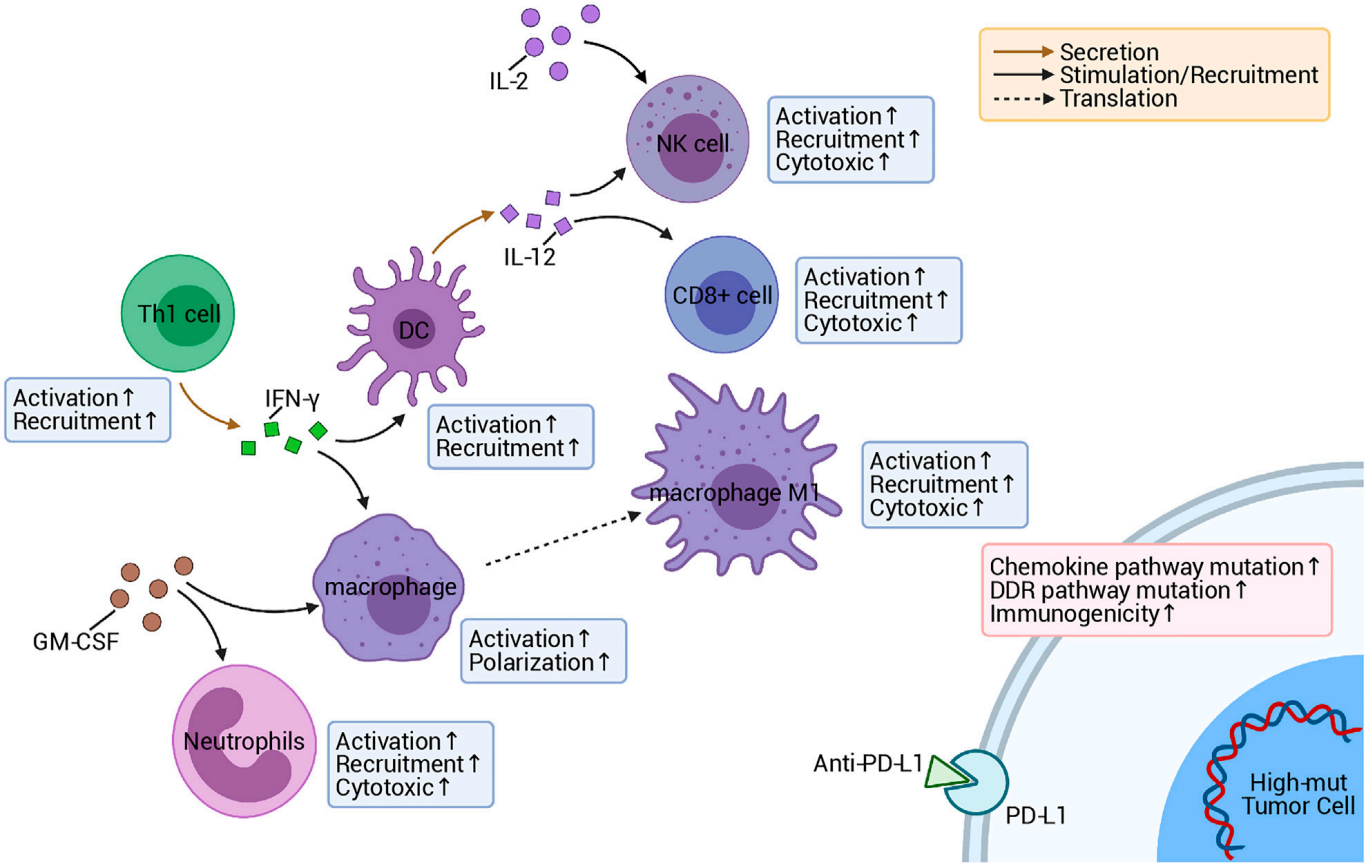
Possible mechanism underlying the improved responses and prognosis of patients with high-mut tumors receiving ICI.

In this research, we noticed that chemokine pathway mutations can be used as a predictor of an improved prognosis of patients after treatment with ICI, based on clinical data and somatic mutation data of a COAD immunotherapy cohort (Figure 1A). In addition, the high-mut patients had a significantly higher OS than the low-mut patients (Figure 1B). Subsequently, we found that the expression of immune checkpoint molecules, which are an important means for tumors to escape immune surveillance and an important marker to predict the prognosis of immunotherapy (Darvin et al., 2018), was higher in high-mut patients. The immune checkpoint genes with high expression levels are often an indicator of a better prognosis following immunotherapy. These conclusions support our hypothesis that the counts of non-synonymous mutations in chemokine pathway can serve as a potential biomarkers to predict the prognosis of COAD patients undergoing treatment with ICI (Figure 8).

High immunogenicity is also beneficial in assisting the immune system to recognize tumor cells and further improve the prognosis of immunotherapy (Wang et al., 2019). With further research into immunogenicity, we can better clarify the reasons for the better prognosis of high-mut patients in response to ICI treatment. In this research, we compared the differences in immunogenicity between high- and low-mut groups regarding TMB, NAL, and DDR pathway, and MSI level, and found that TMB, NAL, and MSI scores, and the number of DDR mutations in the high-mut group were significantly higher than those in the low-mut group. TMB, as the latest biomarker for evaluating ICI treatment in many studies (Choucair et al., 2020), has been proven to be effective in predicting the prognosis of treatment with anti-PD-1 (Le et al., 2015, 2017). Generally speaking, the higher the TMB, the higher the probability and quantity of tumor-specific antigens (new antigens) produced. The appearance of new antigens being more conducive to the immune system recognizing antigens and improving the effect of immunotherapy (Schumacher and Schreiber, 2015). The DDR pathway is an important pathway for maintaining genomic stability, and an increase in mutations of this pathway results in an increase of TMB, which is closely related to improved clinical results of immunotherapy (Ricciuti et al., 2020). Similarly, MSI-H is related to the prognosis of patients receiving immunotherapy, and its predictive effect on prognosis has been demonstrated in many studies (Luo et al., 2020).

In addition to immunogenicity, TME is also an major factor influencing the prognosis of immunotherapy patients. The investigation of TME can help us to further discover the mechanism of how the chemokine pathway is beneficial to the prognosis of high-mut patients receiving immunotherapy. Therefore, we studied the proportion of 22 types of immune cells in patients’ TME using CIBERSORT (Newman et al., 2015). It was found that the TME of patients with high-mut were significantly infiltrated with, activated and related tumor killing tumor cells, such as M1 macrophages, neutrophils and activated NK cells. The degree of infiltration of these cells also showed a positive correlation with the number of chemokine pathway mutations. Macrophages have two phenotypes, M1 and M2, among which M1 cells are pro-inflammatory cells, producing a variety of inflammatory cytokines, such as IL-6, IL-12, and TNF-α, which play an important role in anti-tumor immunity (Shapouri-Moghaddam et al., 2018). NK cells have a cytolytic function, and some studies have proven that the activity of NK cells can determine the effect of anti-PD-1/PD-L1 therapy (Hsu et al., 2018). In contrast, neutrophils generally have two opposing functions, playing both an anti-tumor and tumor-promoting role (Powell and Huttenlocher, 2016). Interestingly, in colorectal cancer, neutrophils show more of an anti-tumor effect (Berry et al., 2017). Our GSEA analysis also showed that the pathways related to killing tumor cells were significantly upregulated in the high-mut group. It is worth mentioning that some classical immune-related pathways, such as JAK-STAT, Toll-like receptor, and Fc-γ receptor, are also upregulated in the high-mut group, and these pathways are closely related to the activation of immune cells. For example, JAK-STAT contributes to the expression of M1 macrophages (Lawrence and Natoli, 2011), and can promote the activation of NK cells (Gotthardt and Sexl, 2016). Toll-like receptor pathways hold an important role in early immune response and macrophage polarization (Zeng and Jewell, 2019). Fc-γ receptors are expressed in a variety of immune cells, and can activate the cytotoxicity of NK cells, macrophages, and other immune cells (Masuda et al., 2009). These findings are helpful in explaining the mechanism of the difference in immune cell infiltration between high- and low-mut groups.

Cytokines are also an important means of regulating the immune response, and as cytokine-related pathways are highly activate in high-mut patients, this may be the molecular basis for why these patients benefit from ICI treatment. Previous studies have shown that the tandem connection of IL-12 and IFN-γ can promote the anti-PD-1 anti-tumor reaction, and the activated atypical NF-kappa B pathway can also promote the production of IL-12 (Garris et al., 2018). The results are consistent with our GSEA analysis results, which showed that leukocyte activation is involved in the inflammatory response, positive regulation of NK cell mediated immunity, positive regulation of interferon-gamma production, positive regulation of interleukin-12 production, and the NF-kappa B signaling pathway. In addition, regulation of granulocyte macrophage colony-stimulating factor production pathway and chemokine receptors bind chemokines pathway are upregulated, which indicates that granulocyte-macrophage colony stimulating factor and chemokines may also promote the proliferation and activation of immune cells and enhance the immune response of the high-mut group (Mellstedt et al., 1999; Griffith et al., 2014; Sokol and Luster, 2015; Ushach and Zlotnik, 2016). Pathways related to fatty acid metabolism and transport, which are connected to immune exhaustion and tumor metastasis (Kim et al., 2019; Zhao et al., 2019), were significantly down-regulated in the high-mut group.

This study still has some limitations. Only one COAD cohort treated with ICI was included in the study. This is because there is a lack of data in this field. There is only one COAD data treated by ICI, but this will not affect our conclusion, because we have found similar phenomena in other cancers (Figures 7B–D), indicating that our research is not accidental. Of course, we also hope to collect more COAD cohorts treated with ICI in the future to further verify our results and expand our results to other cancers. In addition, we did not combine chemokine mutation status with TMB or MSI score for survival analysis, because the number of subgroups was not statistically significant due to insufficient data, but our Multivariate Cox regression analysis proved that chemokine was an independent risk factor (Figure 1B). For this analysis, we will supplement it when the sample size is larger in the future. In addition, there are too many sites of chemokine signaling mutation, so the mechanism of mutation affecting TME is not clear. However, we compared them with the wild type after they mutated, and speculated in combination with the analysis results of GSEA and the results of some previous basic experiments, which is helpful for the future mechanism research.

## Conclusion

In this study, we found that the mutation status of chemokine pathways is closely related to the prognosis of COAD patients being treated with ICI. In addition, that patients with a high-mut status are the dominant population of those receiving ICI treatment, and have a significantly higher OS compared to the low-mut patients. The higher the number of TMB, NAL, and DDR mutations, and presence of inflammatory TME may be the mechanism behind the better prognosis. The discovery of this new biomarker has great potential value in predicting the efficacy of treating COAD patients with ICI. It provides a novel idea for the future of treating cancer patients with ICI.

## Data Availability

The original contributions presented in the study are included in the article/Supplementary Material, further inquiries can be directed to the corresponding authors.

## Supplementary Material

Supplementary Figure S1 Results of the computation/exclusivity analysis of the top 20 mutated genes in the ICI-treated cohort (Samstein et al., 2019) (A) and TCGA-COAD (B) cohort.

Supplementary Table S1 Results of Connectivity Map analysis of the 500 highest and lowest regulated differentially expressed genes in the TCGA-COAD cohort.

Supplementary Table S2 The genes in chemokine pathway.
